# Determination of medication adherence and related factors among people living with HIV/AIDS in a Turkish university hospital

**DOI:** 10.3906/sag-1802-137

**Published:** 2019-02-11

**Authors:** Erdal CEYLAN, Ayşegül KOÇ, Ahmet Çağkan İNKAYA, Serhat ÜNAL

**Affiliations:** 1 Department of Nursing, Faculty of Health Sciences, Ankara Yıldırım Beyazıt University, Ankara Turkey; 2 Department of Infectious Diseases, Faculty of Medicine, Hacettepe University, Ankara Turkey

**Keywords:** HIV/AIDS, antiretroviral treatment, medication adherence, nursing

## Abstract

**Background/aim:**

Widespread use of antiretroviral treatment (ART) has led to decrease in the incidence of HIV/AIDS-related mortality. Besides the availability of ART, medication adherence is essential for treatment success. There is a scarcity of data reported from Turkey regarding ART adherence among people living with HIV/AIDS (PLWHA). Therefore, this study was undertaken to determine medication adherence and related factors among PLWHA in Turkey.

**Materials and methods:**

The sample consisted of 158 PLWHA, who were being followed up at Infectious Diseases Outpatient Clinic of Hacettepe University Hospital. Data were collected using an individual questionnaire and the Turkish version of the Morisky Medication Adherence Scale.

**Results:**

The median patient age was 38 years, 80.4% were male, and 51.3% were married. The median duration of both HIV infection and ART was 3 years. Sixty-one percent used two drug regimens. Sixty-one percent were highly adherent to ART while 37.9% were moderately adherent. The absence/presence of social support resources, disease duration, ART duration, and being informed about the ART regimen were statistically associated with medication adherence.

**Conclusion:**

Our results suggest that medication adherence is excellent among Turkish PLWHA. Interventions, including effective social support, and continuous counseling about ART, might further boost the adherence of PLWHA.

## 1. Introduction

There are 36.9 million people living with HIV/AIDS (PLWHA) in the world. Among them, 34.3 million are adults, and 17.4 million are women (1). According to the Turkish Ministry of Health’s National HIV Registry, about 17,000 people are living with the HIV infection in Turkey. However, due to the long latency, lack of adequate target population testing, and inadequate registry systems, this number may not be robust (2). 

From 2001 to 2013, the annual number of new HIV infections declined by 38% globally, followed by a significant decline in AIDS-related deaths. However, trends in new infections have differed among regions and countries (3), and the number of new HIV diagnoses increased 450% after 2012 in Turkey (2).

There is no cure for HIV infection. Nonetheless, antiretroviral treatment (ART) can be implemented to overcome the infection-associated adverse outcomes (4,5). ART is the cornerstone of management of PLWHA. Widespread use of ART led to a decrease in the incidence of HIV/AIDS-related mortality both in the developed and developing world (6). Furthermore, ART improves the quality of life and prolongs the life expectancy (7).

Medication adherence is defined as ‘‘the act of conforming to the recommendations made by the provider with respect to timing, dosage, and frequency of medication taking’’ (8). Besides the availability of ART, medication adherence must be at least 90% to suppress HIV replication (9–11). Medication adherence is crucial in maintaining therapeutic drug levels, ensuring virologic suppression, reducing drug resistance (12), preventing immunologic decay from HIV, and, especially, reducing the risk of HIV transmission (13). 

Variation in medication adherence has been reported among PLWHA by various studies. Data from Georgia, Iran, Russia, and Greece suggested that the proportion of the adherent PLWHA was 79.0%, 63.9%, 82.0%, and 81.8%, respectively (14–17). A small and older study composed of 36 PLWHA from Turkey found the ART adherence rate as 38.9% (18). 

Risk factors associated with low medication adherence were being busy, forgetfulness (19,20), side effects of ART, economic problems (8), stigmatization (8,21,22), and emotional stress (23,24). In addition, lack of central insurance and therefore high cost is another reason for low adherence (25). In Turkey, it is stated in the 67th article of the Social Insurance and General Health Insurance Law that people with any communicable diseases can benefit from the General Health Insurance system without any delay. HIV infection is also classified in this list. Hence, ART and other medical costs are fully covered by the government (26). PLWHA are charged only for a small amount of the expenditure per prescription. It is called the “contribution margin” and is 10% of drug price (27).

It is important to evaluate ART adherence among PLWHA. There is a scarcity of data reported from Turkey regarding ART adherence. This study was undertaken to determine the medication adherence and related factors among PLWHA in Turkey.

## 2. Materials and methods

### 2.1. Patients

This hospital-based descriptive study was conducted at the HIV/AIDS Outpatient Clinic of Hacettepe University Hospital in Ankara between 7 September 2015 and 27 April 2016. Hacettepe University Non-invasive Ethical Research Board Study reviewed and approved the study protocol (Ref. No.: 16969557-981–02.09.2016). The researchers worked in collaboration with the clinicians in the identification of HIV/AIDS patients, who were receiving ART. A written statement was also included in the introductory part of the questionnaires, explaining the study purpose and confidentiality of the information collected. All data were obtained from respondents in a private room. 

A total 310 PLWHA were being followed up at the HIV/AIDS outpatient clinic. The calculated sample size was 138, assuming α = 0.05 and 1-β = 0.95 in power analysis. During the study period, 186 PLWHA applied to the infectious diseases clinic for treatment and follow-up. Among them, 158 PLWHA who fulfilled the inclusion criteria and voluntarily agreed to participate in the research were included in our study. The inclusion criteria were to be on ART for at least 6 months, at least 18 years old, and to provide informed consent. Exclusion criteria included not providing informed consent and not being on ART. A total of 28 patients were excluded, as they did not meet the inclusion criteria. As a result, 85% (n = 158) of the PLWHA who applied to the infectious diseases clinic were included in the final analysis (Table 1). Our results of the 158 study participants are representative of 310 PLWHA treated in the HIV/AIDS outpatient clinic.

**Table 1:** Distribution of the adherent and noncompliant people living with HIV/AIDS according to descriptive statistics of demographic variables.

				Adherence level		
				High n (%)	Medium/Low n (%)	P
Age			18–25	11 (64.7)	6 (35.3)	0.956
			26–45	57 (62.0)	35 (38.0)	
			46–60	23 (60.5)	15 (39.5)	
			>60	6 (54.5)	5 (45.5)	
Sex			Male	79 (62.2)	48 (37.8)	0.988
			Female	18 (60.0)	12 (40.0)	
Marital status			Married	49 (60.5)	32 (39.5)	0.812
			Single	48 (62.3)	29 (37.7)	
Number of drugs taken		One-drug regimen		17 (58.6)	12 (41.4)	0.464
		Two-drugs regimen		63 (64.9)	34 (35.1)	
		Three or more drugs		17 (53.1)	15 (46.9)	
Education	Primary school and below			12 (54.5)	10 (45.5)	0.181
	Secondary school			9 (45.0)	11 (55.0)	
	High school			17 (56.7)	13 (43.3)	
	Undergraduate and master degree			59 (68.6)	27 (31.4)	

### 2.2. Data collection 

Data were collected using an individual information questionnaire and the Turkish version of the Morisky Medication Adherence Scale (MMAS). The personal information questionnaire was developed by the researchers, using PubMed and Elsevier databases (8,19,28,29). This questionnaire included 7 questions about the participant’s demographic characteristics (sex, age, marital status, occupation, education level, social insurance, and economical status) and 22 questions on medication and disease-related factors (disease and medication duration, name and number of pills taken, patients’ thoughts about the benefits, complexity, taste, and number of the drugs; reasons for missing the doses, such as being busy, side effects, vision problems, economic problems, stigma, lack of social and health care professionals’ supports; methods used as a reminder of the dose time, and assessing if the patients had any information about the usage of the drugs) 

The Turkish version of the MMAS was used to determine adherence to ART among PLWHA. The reliability and validity studies of the Turkish version of the MMAS were previously reported by Vural et al. (30). This scale consists of 6 items, each being a 2-point type, implemented in evaluating ART compliance through a face-to-face interview. Higher scores indicate lower adherence to ART. According to this scale, zero score implies high adherence, 1–2 points denote moderate adherence, and 3–6 points means noncompliant. 

After informing the patient about the study, the data report form was distributed, and correspondents asked to fill the questionnaire in a private room located at the outpatient clinic, by paying attention to their privacy. 

### 2.3. Statistical analysis

All analyses were performed using SPSS version 21.0 (IBM Corp. Released 2012. IBM SPSS Statistics for Windows, Armonk, NY, USA: IBM Corp.) and Microsoft Excel 2013. Numbers, percentages, median, and the chi-square (χ2) test were used in the data analysis. The Shapiro–Wilk test was used to determine whether the data were normally distributed. Spearman’s rho coefficient (*r*s) allowed identifying whether there was an association between age/duration of illness/duration of medication/number of medicines used/daily dose amount, and the MMAS score. The Mann–Whitney U and Kruskal–Wallis tests were adopted to determine whether there was a statistically significant difference in the MMAS score among the groups (female/male, marital status, education level, having/not having information about drugs, experiencing/not experiencing side effects, presence/absence of social support, being satisfied/dissatisfied with the healthcare professionals, stigma, use/do not use reminders). The statistical significance level was set at P < 0.05.

## 3. Results

There were 500 PLWHA registered in the Hacettepe University cohort. Among them, 310 were actively under regular follow-up. One hundred and eighty-six PLWHA applied for follow-up during the study period. Twenty-eight PLWHA were excluded, and 158 PLWHA included in the final analysis (Figure). The median age was 38 (min: 19, max: 74), 80.4% (n = 127) were male, and 51.3% (n = 81) were married. The median duration of both HIV infection diagnosis and ART were 3 years. Sixty-one percent (n = 97) used two drug regimens. Table 1 summarizes the demographic values.

**Figure 1:**
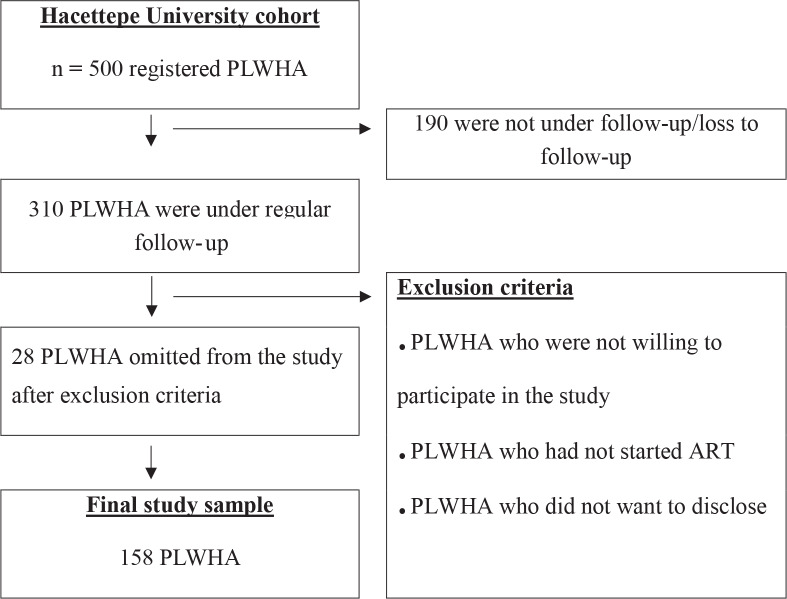
Sampling process of the study (PLWHA, people living with HIV/AIDS; ART, antiretroviral treatment)

Nearly all the respondents (98.7%) thought that the drugs are beneficial for them, but 26.6% (n = 42) reported that they missed at least one dose because of being busy. In addition, 28.5% (n = 45) thought that the number of the pills taken daily are too many, and 29.7% (n = 47) had ART-related side effects. Also, 8.2% (n = 13) had problems affording the drugs, and 17.1% (n = 27) did not have information about the importance of adherence to ART and how to use the drugs. Furthermore, 42.4% (n = 67) had stigma-related problems.

Sixty-one percent (n = 97) were highly adherent to ART while 37.9% (n = 60) were moderately adherent. Adherence varied among the age groups. Adherent PLWHA was 64.7%, 62.0%, 60.5%, and 54.5% in the age groups 18–25, 26–45, 46–60, and over 60 years, respectively. There were no significant differences in adherence among the age groups (χ2 = 0.321; P = 0.956). Medication adherence was not associated with sex, age, marital status, education level, and the number of prescribed drugs in the ART regimen (P > 0.05) (Table 1).

Medication adherence differed in PLWHA who were previously informed about ART and those who were not. Adherence among the preinformed and uninformed patients was 65.6% and 40.7%, respectively (Table 2). Those who had information on the usage and importance of ART were statistically more adherent than those who did not have such information (χ2 = 4.856; P = 0.028). 

**Table 2:** Distribution of the adherent and noncompliant people living with HIV/AIDS, according to factors related to the disease.

Factor		Adherence level		
		High n (%)	Medium/Low n (%)	P
Having information about ART	Yes	86 (65.6)	45 (34.4)	0.028
	No	11 (40.7)	16 (59.3)	
Having side effects due to ART	Yes	28 (59.6)	19 (40.4)	0.899
	No	69 (62.2)	42 (37.8)	
Having social support resources	Yes	92 (64.8)	50 (35.2)	0.019
	No	5 (31.3)	11 (68.8)	
Being satisfied with healthcare professionals’ attitudes	Yes	94 (61.8)	58 (38.2)	0.677
	No	3 (50.0)	3 (50.0)	
Stigma	Yes	43 (64.2)	24 (35.8)	0.537
	No	54 (59.3)	37 (40.7)	
Using reminders	Yes	38 (66.7)	19 (33.3)	0.394
	No	59 (58.4)	42 (41.6)	
Having financial problems	Yes	8 (61.5)	5 (38.5)	1.000
	No	89 (61.4)	56 (38.6)	

The presence of ART-related side effects did not affect adherence. There was no difference in medication adherence between patients with and without side effects (59.6% and 62.2%, respectively) (χ2 = 0.016; P = 0.899) (Table 2). Conversely, a significant association existed between the presence of social support and medication adherence. PLWHA who confirmed the presence of social support were more adherent than the PLWHA who did not have social support (64.8% and 31.3%, respectively) (χ2 = 5.483; P = 0.019) (Table 2). 

The relationships of the MMAS score with age, duration after diagnosis, duration of ART, number of medications prescribed, and number of pills taken daily were examined. A low positive correlation between duration after diagnosis, duration of ART, and the MMAS score was demonstrated (*r*s = 0.275 and 0.254, P ≤ 0.001, respectively) (Table 3).

**Table 3:** Correlation between the Morisky Medication Adherence Scale score and selected variables

Variable	Morisky adherence score	
	r_s_	P
Age	0.028	0.724
Duration of disease	0.275	<0.001
Duration of drug usage	0.254	0.001
Number of drugs taken	0.068	0.393
Number of pills taken daily	0.134	0.094

## 4. Discussion

Our results revealed that 61% of PLWHA were adherent to ART, whereas 1.3% were noncompliant. The adherence rate in this study was higher when compared with the reported rates in the literature. It was previously shown that 21% to 94% of PLWHA were highly adherent to ART (15,17,29,31­–35). The variance in adherence rates may result from differences in the research method, characteristics of the cohort, the administrative structure of the healthcare facilities, and the social security policies of the countries. It may also arise from the economic status of the patients, because people living in Europe, Asia, Africa, and America have different socioeconomic levels.

In previous studies, ART adherence was associated with sex (23), age (36), education (19,22), economic status (8,24,25,33), substance use (19,23,25), disease duration (37), stigma (8,22,23,33,38), ART-related side effects (8,38–41), satisfaction with healthcare staff (8,33,38,39), number of pills taken daily (21,31,32,42), social support (8,38,43), mental health (23–25,43), using reminders (8,43), and having information prior to ART (38). In addition, being busy, forgetfulness (19,28,44), being away from home (41,44), lack of central insurance (25,43), and adverse taste of medicines (45) were the most common reasons for low adherence. After examining the reasons for low adherence, according to the countries, the most leading causes included being busy, being away from home, and forgetfulness, in the USA; side effects, substance abuse, and stigma in Europe; stigma, forgetfulness, and substance abuse in Asia; and lastly, economic status, side effects, satisfaction with healthcare staff, and stigma in Africa. Mental health was the common factor that was associated with ART adherence in all regions. In our study, no correlations were observed between ART adherence and age, sex, marital status, education level, economic status, stigma, ART-related side effects, satisfaction with healthcare staff, and the number of pills taken daily. The fact that our sample is small, that the study is single-centered, and that we work at the referral center for HIV/AIDS may have affected these results. In addition, the small number of people with low adherence-related risk factors, such as lack of health insurance, dissatisfaction with healthcare personnel, and economic problems, may have contributed to these results.

In this study, people who were previously informed about ART adherence had higher adherence rates compared to those who were uninformed. Our results agree with those of Schneider et al. who demonstrated a statistically significant relationship between having information about ART and ART adherence (46). Similarly, Demirkıran et al. (47) found that informing patients about medication adherence improves adherence among PLWHA. Providing information to PLWHA on ART and adherence ensures taking medicine regularly, without missing any dose, despite feeling good/bad, having side effects, being bored of the drugs, or feeling no need to continue the medication; thereby maintaining the high ART adherence level. Furthermore, in a routine daily outpatient care, every single PLWHA applying to our clinic was informed about ART and the disease itself. Interestingly, some of the PLWHA under our care were still denying previous education on ART and the disease. This finding restresses the importance of continuous medical briefing at every visit. 

Another factor that was significantly associated with ART adherence was the absence/presence of social support resources (Table 2) (P = 0.019). Kalichman et al. (48) observed that patients who had lack of support resources were less adherent to ART. In another study, in which pharmacists talked about their experience while working with PLWHA, lack of support resources was the major obstacle for ART adherence (25). Our findings are consistent with those of the previous studies in the literature. The absence of social support increases the feelings of anger, offense, despair, and depression in the later stages of HIV/AIDS infection. Consequently, all of these factors affect patients’ adherence to ART adversely. 

No statistical association was found between ART adherence and having ART-related side effects, being satisfied/dissatisfied about the healthcare professionals, stigma, using reminders, and economic status (P > 0.05) (Table 2). In contrast to our findings, PLWHA who have ART drug-related side effects (8,20,36,37,49), are dissatisfied about health care professionals (50) and had stigma-related problems (8,21,22) were found to be less adherent in other studies. From this perspective, our findings were inconsistent with those in the literature. These discrepancies may be because most of our respondents were satisfied with the healthcare professionals in the HIV/AIDS Outpatient Clinic. Also, the prescribed ART was current, and therefore most of the PLWHA did not experience ART-related side effects. Furthermore, we did not use a comprehensive tool to evaluate the stigma. Instead, it was asked with only one question in the questionnaire form. These reasons may explain the lack of association between stigma, ART-related side effects, being satisfied/dissatisfied with the healthcare professionals, and ART adherence.

In our study, weak positive correlations were observed between disease duration, ART duration, and ART adherence (P = 0.001) (Table 3). It was found that taking ART for a long time could increase the motivation for medication adherence. Our findings corroborated the literature (36,51). When the duration of ART is long, PLWHA become used to the drugs, require fewer reminders, and the drugs become a regular part of their lives. All these factors are thought to maintain a high medication adherence among PLWHA.

This work is the first extensive study from Turkey, determining ART adherence and related factors. Our study was undertaken in a tertiary level referral center, with strict case and outcome definition criteria. The prospective study design minimized the missing data. Our sample size is statistically sufficient to draw a conclusion on the ART adherence among Turkish PLWHA. Conversely, the single-center design, lack of a reliable stigma scale, and lack of pharmacy refill dates are main limitations in our study.

In conclusion, the absence/presence of social support resources, disease duration, ART duration, and being informed about the ART regimen were statistically associated with medication adherence. Therefore, PLWHA must be continuously informed about the ART daily dosing regimen, as an intervention to maintain a high ART adherence. Suggesting reminders, ensuring and searching social support resources, and determining the factors that negatively affect ART adherence represent the other solutions and propositions for increasing ART adherence within the framework of nursing. Finally, considering that newly diagnosed patients and those who are new to ART have relatively low drug compliance, informing PLWHA on the use of ART should be further intensified in newly diagnosed individuals and those who have just started ART.

## Acknowledgments

This study was supported by a grant from the Yıldırım Beyazıt University Scientific Research Department (Project No.: 3049).
